# Measuring Mindfulness in Business School Students: A Comparative Analysis of Mindful Attention Awareness Scale and Langer’s Scale

**DOI:** 10.3390/bs13020116

**Published:** 2023-01-30

**Authors:** Mohammed Laeequddin, K. Abdul Waheed, Vinita Sahay

**Affiliations:** 1 Indian Institute of Management Bodh Gaya, Bodh Gaya 824234, India; 2 Shiv Nadar Institution of Eminence Deemed to be University, Greater Noida 201314, India

**Keywords:** mindfulness, measuring mindfulness, mindful attention awareness scale, Langer’s mindfulness/mindlessness scale

## Abstract

Research studies have established that mindfulness helps in psychological well-being, stress reduction, chronic pain management, behavioral therapy, and other areas including organizational development. Mindfulness often refers to a state of consciousness, but mindfulness can also be understood as a personality trait. State mindfulness is referred as the individual’s capacity to cultivate a particular state of mind during meditative practice. Traits are more permanent facets of personality characteristics that are difficult to change and likely have some basis in genetics. Few scholars have criticized meditative mindfulness as a trend and cautioned that organizations should carefully consider their goals before introducing meditative mindfulness training. This dichotomy has prompted us to review the literature and carry out a comparative analysis of two divergent measurement scales of mindfulness: the Mindful Attention Awareness Scale (MAAS) and Langer’s mindfulness/mindlessness scale. The MAAS is the most widely used mindfulness scale to measure mindfulness, and Langer’s scale measures mindfulness/mindlessness. We developed hypotheses relating Langer’s scale and the MAAS. Further, we studied whether there is any difference in mindfulness/mindlessness among business school students with an undergraduate background in engineering and nonengineering streams. Using a self-administered questionnaire, we measured the mindfulness levels of 221 MBA first- and second-year graduates and tested the hypothesis using partial least squares structural equations modeling (PLS-SEM). We found that Langer’s mindfulness/mindlessness scale was negatively associated with the MAAS. We did not find any effect of gender, education, and professional specializations on mindfulness.

## 1. Introduction

Over the last decade, both clinical and nonclinical researchers have started to pay a significant amount of attention to mindfulness research. It has been established that mindfulness helps with psychological well-being, stress reduction, chronic pain management, behavioral therapy, and other areas. Some studies claimed that in organizations, mindfulness is used as a means for reflection and learning [1], human resource practice [2], self-awareness, the emotion regulation of leaders [3], developing leaders [4], and the remote engagement of employees to address the new normal [5]. The psychological perspective on mindfulness emerged in 1979 when Kabat-Zinn integrated Buddhist mindfulness meditation into clinical and psychological practice in a stress reduction and relaxation program called Mindfulness-Based Stress Reduction (MBSR) to relieve suffering among patients with chronic pain and facilitate adaptation to medical illness. Ref. [6] defined mindfulness as “awareness that arises through paying attention with purpose, non-judgmentally, while being in the present moment”. This definition describes that awareness arises when one pays attention to an object with the intention to regulate attention(purpose) to sustain the action of attention without judging the experiences while paying attention and being in that moment. Some scholars have criticized the practice of meditative mindfulness as a superficial trend. They view meditative mindfulness as a dramatic method of promoting the meditative practice that is based on claims that are questionable [7,8,9]. Ref. [10] caution that organizations should carefully consider their goals before introducing meditative mindfulness training. Ref. [11] argued that self-control exercises might be used to increase the self-regulatory capacities of individuals. Ref. [12] questioned the beliefs in favor of the benefits of self-regulation exercises, loving–kindness meditation, and progressive muscle relaxation. Ref. [13] argued that mindfulness is “a universal human capacity that need not be enhanced through the practice of meditation; instead, mindfulness is gained by maintaining orientation in the present, openness to novelty, alertness to distinctions, sensitivity to different contexts, and an awareness of multiple perspectives”. Langer’s construct of mindfulness essentially underlies the several components of creativity [14] and needs to be empirically mapped to contemplative conceptualizations of mindfulness [15]. While meditative mindfulness helps individuals practice their minds to enhance their attention and observation skills [16], Langer’s conceptualization of mindfulness and its related interventions can be considered a cognitive task [14]. 

This difference in the two divergent conceptualizations of mindfulness in the extant literature drew us to empirically investigate the relationship between them. Further, we measured these two conceptualizations of mindfulness in business school students. This paper is organized as follows: The second section presents a literature review on mindfulness and mindfulness measuring scales and hypothesis development. The third section provides the research methodology, the fourth section discusses the findings and data analysis, and the fifth section offers conclusions and directions for further studies.

### 1.1. Literature Review and Hypothesis Development

Mindfulness can be broadly classified from three different perspectives: (i) The Buddhist contemplative perspective. Ref. [17] describes mindfulness as “the deliberate use of thought and concepts to keep the object before the mind instead of allowing thought to drift at random, governed by defiled emotions, habit patterns, and practical survival needs”. (ii) Kabat-Zinn’s cognitive (psychological) perspective. Ref. [18] described mindfulness as “a disciplined awareness, whereby one may take responsibility for each moment experienced, whether the moment is comfort, pain, stress, or joy, as a psychological state that can be cultivated”. Psychology scholars view mindfulness as a mechanism of meditation and argue that meditation works through psychological mechanisms, and it is usually measured with the attention and awareness scale [19]. However, the Buddhist definition and description of mindfulness do not consider meditation or meditative intervention as mindfulness. (iii) Langer’s nonmeditative (creative) mindfulness perspective. Ref. [13] argues that “mindfulness explicitly involves making deliberate cognitive categories, generating new distinctions, and adapting to changing situations.” This concept reflects four interrelated components: (a) novelty seeking, (b) engagement, (c) novelty producing, and (d) flexibility.

Ref. [20] said that mindfulness often refers to a state of consciousness, but mindfulness can also be understood as a personality trait. State mindfulness is described as the attentive metacognitive monitoring of present emotions, perceptions, and sensations [21]. A state refers to a fluid and short-term mindset or frame of reference that we can quickly move in and out of, sometimes by force of will. It is a flexible condition that influences how we perceive the world around us. Ref. [22] referred to state mindfulness as the individual’s capacity to cultivate a particular state of mind during meditative practice. Trait mindfulness refers to an individual’s tendency to display a lack of judgement and open awareness in their emotions and actions in day-to-day life [23]. However, traits are more permanent facets of personality, characteristics that are difficult to change and likely have some basis in genetics. The Western concept of mindfulness (Langerian concept) refers to mindfulness as a stable trait [24]. Kabat-Zin’s concept is often referred to as the Eastern concept of mindfulness. Ref. [25] mentioned that some people may be in a mindful state of consciousness more often than others due to differences in an individual’s dispositional tendencies. Individuals with high trait mindfulness will be in a state of consciousness with their attention being focused on present-moment phenomena occurring both externally and internally. Trait mindfulness refers to the duration, frequency, and intensity with which an individual tends to engage in states of mindfulness [26], whereby those with greater emotional stability [27] and more adaptive responding to negative events [28] may perceive difficult situation as less stressful. Ref. [19] mentioned that trait and state mindfulness are independently associated with lower levels of negative affect in daily life.

Historically, mindfulness has been conceptualized as a state of consciousness (“state mindfulness”) that is achieved through meditation [29] or some other intervention such as Mindfulness-Based Stress Reduction [30]. According to [31], trait mindfulness refers to a person’s propensity to practice mindfulness in daily life even in the absence of any interventions. They also claimed that this propensity typically persists over time. Therefore, we can infer that the trait’s permanent attributes to personality characteristics that may not change generally have some basis in genetics. Ref. [32] described a trait of individual personality as a relatively continuous pattern of thought, feeling, and behavior patterns that distinguish one person from another.

#### 1.1.1. Association between Langerian and MAAS Measurement of Mindfulness

Mindfulness is measured either by neuroimaging or self-reporting. Recently, various self-reporting scales have been developed to measure the state and trait mindfulness of meditating and nonmeditating targets [33]. The complexity is due to the terminological and conceptual confusion of researchers approaching the subject from different perspectives [34]. Most of the scales measure trait mindfulness of meditators and nonmeditators alike, considering mindfulness as an intrapsych process. The extant literature presents eight mindfulness questionnaires that have been validated: (1) the Freiburg Mindfulness Inventory (FMI) [35], which measures mindful presence and mind/body awareness; (2) the Mindful Attention Awareness Scale (MAAS) [19], which measures the presence of mind; (3) the Cognitive and Affective Mindfulness Scale-Revised (CAMS-R) [36], which measures attention, present focus, awareness, and the acceptance/nonjudgment of thoughts and feelings; (4) the Southampton Mindfulness Questionnaire (SMQ) [37], which measures accepting difficult thoughts/images and oneself versus judging cognitions and the self; (5) the Kentucky Inventory of Mindfulness Scale (KIMS) [38], which measures acceptance without judgment; (6) the Five Facet Mindfulness Questionnaire (FFMQ) [31], which measures nonjudgement; (7) the Philadelphia Mindfulness Scale (PHLMS) [39], which measures acceptance; and (8) the Toronto Mindfulness Scale (TMS) [40] which measures curiosity.

The MAAS has been very popular for measuring mindfulness as it assesses how individuals pay or do not pay attention to their thoughts, feelings, physical sensations, and tasks [41]. Ref. [19] found that MAAS scores were significantly higher among meditation practitioners relative to nonpractitioners. The MAAS has strong measurement properties with a stable unidimensional structure, and the scale has been tested by different research groups across the globe, resulting in the finding that the unidimensional structure of the construct is intact [42]. The scale is suitable for both individuals who have had meditation experiences and those who have not had meditation experiences. Furthermore, the MAAS is a commonly used scale of mindfulness as it is a very concise and well-validated instrument that can be used both in clinical and nonclinical populations [43]. The scale includes 15 Likert scale items and six categories and ranges from 1 (almost always) to 6 (almost never). It evaluates the general disposition to be mindless and on auto-pilot mode (trait mindfulness) and thus defines mindfulness as the absence of mindlessness [44]. The mean of all responses is taken as a total score, whereby a higher score represents a greater mindfulness level. The MAAS assumes that one can be mindful when he/she is in a controlled state in which one is asked to notice one’s inner experiences or thoughts. Though some scholars (e.g., [45]) consider the MAAS as a trait mindfulness scale, the MAAS mainly measures nonjudgmental attitudes instead of measuring mindfulness as described by [17], who conceptualized mindfulness as “the deliberate use of thought to keep the object before the mind instead of allowing thought to drift at random governed by defiled emotions, habit patterns”. 

Contradicting the conceptualization around the MAAS, Ref. [13]’s mindfulness explicitly assesses the sociocognitive approach to mindfulness that differs from the meditative approach. It usually includes the external, material, and social context of individual participants [13,46]. Ref. [47] described mindfulness as “when in a mindless state, an individual operates much like a robot; thoughts, emotions, and behaviors are determined by ‘programmed’ routines based on distinctions and associations learned in the past”, which is auto pilot or trait mindfulness. Ref. [13]’s model of mindfulness focuses on how individuals generally interact with their environments. She projected mindfulness as a bipolar continuum with mindlessness and mindfulness at the two extremes. Ref. [13] defined mindlessness as a state of an absence of or decrease in mindfulness. The framework of Langer’s theory of mindfulness has been investigated in the past 25 years. Ref. [48] developed a seven-point 14-item Likert-scale-type Langerian Mindfulness Scale (LMS14) measuring three components (novelty seeking, novelty producing, and engagement) of sociocognitive mindfulness. Ref. [49] stated that the Langer’s Mindfulness scale does not measure "who is or is not mindful at any given time, but rather who is more or less likely to be predisposed to be mindful at any given time”. Although both the MAAS and Langer’s scale measure the trait mindfulness of individuals, we assume that various mindfulness scales may not actually measure the same perspective of mindfulness.

Ref. [31] reported that MAAS scores were found to be correlated with other different measures of mindfulness, mostly in those respondents who had been practicing meditation as a mindfulness intervention. Ref. [31] developed the Five Facet Mindfulness Questionnaire (FFMQ), which measures five elements of mindfulness: observing, describing, acting with awareness, not judging inner experience, and not reacting to inner experience. The questionnaire consists of 39 composite items from five instruments: the FMI, MAAS, KIMS, SMQ, and CAMS. Ref. [31] explored the facet structure of mindfulness by taking the select questionnaires and studied how these facets associate with other constructs such as openness to experience, emotional intelligence, experience avoidance, neuroticism, and disassociation and found a correlation between them. Though [13] argued that “developing parallel between different types of mindfulness is not warranted as they are derived from different contexts, i.e., cultural and historical”, a few studies have shown a weak-to-moderate positive correlation between the MAAS and other mindfulness measures (e.g., [19,31,50]). Ref. [15] suggested that the different conceptualizations of mindfulness need to be empirically investigated as it helps with mapping one conceptualization with others.

As per Langerian conceptualization, it was claimed to help improve social relationships and aid in assessing the social and relational well-being of individuals, whereby the mindfulness of individuals is increased by noticing new things. In several other studies, researchers found that instructional interventions improved the trait mindfulness (e.g., [51,52,53]). Similarly, Ref. [49] found that mindfulness qualities are a trait phenomenon where people seem to have the same way of viewing their environment across time. Ref. [54] experimented by inducing instructional interventions to respondents, which prompted them to intentionally regulate their mode of thinking and thereby shift or switch from mindlessness to mindfulness. Ref. [55] mentioned that the MAAS lacks items distinctly measuring attention/awareness, but it measures the personality traits of an individual with a general tendency to carry out day-to-day activities mindlessly, which is the inherent tendency of the person. In contrast, Langer measures an individual’s mindfulness/mindlessness personality trait in terms of an external focus on (a) novelty seeking, (b) engagement, (c) novelty producing, and (d) flexibility as trait mindfulness. Hence, Langer’s scale shall have a negative association with the MAAS. Therefore, we draw our first hypothesis as follows:

**H1a.**  *Langer’s Mindfulness Scale is negatively associated with the Mindfulness Attention Awareness Scale (MAAS).*

**H1b.**  *Langer’s Mindlessness Scale is negatively associated with the Mindfulness Attention Awareness Scale (MAAS).*

#### 1.1.2. Moderation Effect of Education Background

Ref. [56] found that the average conscientiousness score for engineers was significantly lower than that of nonengineers, which was at variance with typical depictions of engineers as being higher in conscientiousness. Ref. [57] interpreted lower conscientiousness as reflecting flexibility and creativity. In an intrinsic motivation study, Ref. [58] found that engineers scored higher than nonengineers. Therefore, we propose that the traits of engineering graduates and nonengineering graduates opting for various management specializations would be different, hence their mindfulness. Therefore, we draw our second hypothesis as follows:

**H2a.**  *Having an undergraduate education background will have a moderating effect on the association between Langer’s mindfulness and the MAAS. The negative effect of Langer’s mindfulness on the MAAS will be lower in engineering background students in comparison to nonengineering background students.*

**H2b.**  *Having an undergraduate education background will have a moderating effect on the association between Langer’s mindlessness and the MAAS. The negative effect of Langer’s mindfulness on the MAAS will be lower in engineering background students in comparison to nonengineering background students.*

## 2. Methodology

For this study, we considered the two most popular mindfulness scales, the MAAS [19] and Langer’s 14 items scale [48], and measured the mindfulness levels of 221 MBA first- and second-year graduates at a business school of national importance in India and tested our hypotheses using an analysis of variance (ANOVA) and partial least squares structural equations modeling (PLS-SEM). The questionnaire was designed in two parts: the first part consisted of demographic information such as age, gender, professional qualification, and work experience, and the second part consisted of measures of the MAAS [19] and Langer’s 14-item scale [48].

A survey questionnaire was developed in a google form, combining 15 items of the MAAS and 14 items of the Langer’s scale and incorporating demographic details such as gender, professional qualification, and chosen specialization in the MBA program. Initially, a pilot questionnaire consisting of 36 items (7 demographic plus 15 MAAS and 14 Langer’s scale items) was administered online to a convenience sample of 40 students (20 to 28 years old) containing 16 males and 24 females. The reliability and validity of the items were found to be satisfactory. Therefore, we retained all the items and used the same questionnaire for the final survey. The Google form was floated among 361 students (excluding the pilot batch) on 15 November 2021 to complete the survey voluntarily. Within 48 h, we received 128 responses, and further, we pursued students every week until 12 December 2021. Finally, we received 256 responses, out of which 221 responses were found to be complete in all aspects. We analyzed the data using these responses.

## 3. Analysis and Results

We performed partial least squares structural equations modeling (PLS-SEM) using SmartPLS software [59] to assess the measurement properties of the MAAS and Langer’s mindfulness and mindlessness scale and validate the proposed hypotheses of the study [60]. The results of the analysis of the measurement properties of the scales are presented in Table 1 and Table 2. Table 1 shows the loading of each item on the scale with its respective measurement and each item’s loading with other measures (cross loadings). All the items of the MAAS were found to be loaded together as one construct with a factor loading more than 0.50 except for one item, “I find myself preoccupied with the future or the past”, which was found to have a loading less than 0.50, and this item was removed from further analysis. Similarly, three items of Langer’s scale (“I generate few novel ideas; I make many novel contributions; I seldom notice what other people are up to”) were found to have factor loadings less than 0.50 and were removed from further analysis. Table 1 shows only the items that had factor loadings greater than 0.50 with their respective measurement constructs, and they had cross loadings that were lower than their factor loading.

Table 2 shows the details of the reliability and validity testing of the measurement constructs of the study. To assess the reliability of the measurement constructs, PLS-SEM produces statistics such as Cronbach’s Alpha and composite reliability, which is supposed to be at the minimum cut-off of 0.7 [60]. All the measurement constructs passed the reliability tests by meeting the minimum cut-off of 0.7 (MAAS: Cronbach’s Alpha = 0.92, composite reliability = 0.93; Mindfulness—Langer scale: Cronbach’s Alpha = 0.86, composite reliability = 0.89; Mindlessness—Langer scale: Cronbach’s Alpha = 0.69, composite reliability = 0.81). The measurement constructs were tested for both convergent and discriminant validity. Convergent validity is about how well all the items of the measurement construct behave together as one factor, and discriminant validity is about how well the measurement construct is different from other constructs. The convergent validity is tested with the average variance extracted (AVE) value, which is supposed to meet the minimum cut-off requirement of 0.5 [60]. The measurement constructs used in the study, such as MASS (AVE = 0.51) and Langer scale’s mindfulness (AVE = 0.54) and mindlessness (AVE = 0.52), were found to meet the minimum cut-off requirement of 0.5, thereby establishing convergent validity. To test the discriminant validity of the measurement constructs used in this study, Ref. [61] criterion was used [60]. According to this criterion, the square root of the AVE of each construct should be more than that construct’s correlation with other constructs. Table 2 shows the interconstruct correlations and square root of the AVEs in a diagonal position. The square root of the AVEs of all the constructs exceeded the interconstruct correlations, thereby establishing the discriminant validity of the measurement constructs used in the study.

In PLS-SEM, hypothesis testing was performed by assessing the structural model that included the relationship between independent and dependent variables. The structural model was assessed for explanatory power, predictive relevance, and predictive strength [60]. The results of the structural model are presented in Table 3. The explanatory power was assessed with the R^2^ value, which is supposed to be above 0.20 for a satisfactory explanatory power [60]. The R^2^ of the structural model of this study was 0.329, which means that 32.9% of the variance in the dependent variable MAAS was explained by two independent variables, MFL and MLL, which were included in our study. The predictive relevance was established by the Q^2^ value, which was supposed to exceed the value of zero [60]. The structural model of our study resulted in a Q^2^ value of 0.158, establishing a satisfactory predictive relevance. The predictive strength was assessed by the significance of the path coefficients. Both MFL (path coefficient = −0.151; *p* < 0.05 level) and MLL (path coefficient = −0.524; *p* < 0.01 level) were found to have a statistically significant negative effect on the MAAS, thus supporting H1a and H1b of our study.

To validate the hypotheses relating to the moderation effect of undergraduate (U.G.) education background (whether the background is engineering or nonengineering) on the effect of MFL and MLL on the MAAS, the partial least squares multigroup analysis (PLS-MGA) option provided in SmartPLS 3.0 was used [62]. The PLS-MGA is a nonparametric technique that draws the probability value of the difference in path coefficients through the bootstrapping procedure. The probability value of the path coefficients difference is supposed to be either <0.05 or >0.95 to establish a significant moderation effect of the grouping variable. Our data included two groups of U.G. education backgrounds, one group of students with engineering backgrounds (n = 139) and another with nonengineering backgrounds (n = 82). The results of PLS-MGA are shown in Table 4. The results suggested that there was no significant moderation effect of U.G. education background on the association between MFL and the MAAS (path coefficient difference = −0.005; *p*-value = 0.903) and MLL and the MAAS (path coefficient difference = 0.008; *p*-value = 0.942). Therefore, both H2a and H2b of our study were not supported.

In addition to the assessment of the hypotheses proposed in this study through PLS-SEM, we also checked the effect of demographic variables such as gender, U.G. background, and chosen specialization in the MBA program on the measurement constructs. For this, we performed an analysis of variance (ANOVA) using IBM SPSS software. The measurement constructs, the MAAS, Langer scale—mindfulness, and Langer scale—mindlessness, were included as dependent variables in the ANOVA. The mean of all the valid and reliable items of these constructs as identified through the analysis of measurement properties in PLS-SEM was used for this analysis. The results of these analyses are presented in Table 5 and Table 6. Table 5 shows the results of an ANOVA with the MAAS as a dependent variable. The results suggest that there was no effect of gender on U.G. education background and MBA specialization on the MAAS. 

Table 6 shows the results of an ANOVA with Langer scale—mindfulness and mindlessness as the dependent variables. The results showed that gender and MBA specialization had no effect on both the Langer scale—mindfulness and mindlessness. Regarding the effect of U.G. education background, it was found to have no effect on Langer scale—mindfulness, but it was found to have a significant effect on mindlessness (f value = 3.914; *p*-value < 0.05).

## 4. Discussions and Conclusions

We predicted that Langer’s mindfulness and mindlessness scales would be negatively associated with the Mindfulness Attention Awareness Scale (MAAS) and found a statistically significant negative effect on the MAAS; thus, H1a and H1b were supported in our study. Although both the scales measure the trait mindfulness, the negative association can be due to their internal and external focus/attention being paid moment to moment. The MAAS was developed from a meditative understanding of mindfulness [63]. The MAAS measures the presence or absence of attention and awareness in a particular moment, and it is highly used to measure diagnostic ability and less to measure the cognitive capabilities of an individual [63]. Further, the MAAS measures the inherent personality traits of an individual, whereas Langer’s scale measures an individual’s mindfulness/mindlessness personality trait in terms of external focus. Previous studies found a positive correlation between various scales because they all measure personality traits by focusing on the internal characteristics of a person, such as openness to experience, emotional intelligence, experiential avoidance, neuroticism, and disassociation. For example, with an approach to develop a sociocognitive perspective of mindfulness, ref. [63] adopted Langer’s mindfulness scale and achieved discriminant validity with the MAAS, confirming that these two scales measure different constructs; however, they found a positive correlation between these scales. The negative association between the MASS and Langer’s scale could be attributed to the differences in the trait mindfulness of individuals. The MAAS measures the neuroticism trait of individuals. Neuroticism is the trait disposition to experience negative effects, including a depressed mood and anxiety, and it has also been defined in terms of a lack of self-control. Through a meta-analytic study, ref. [25] concluded that mindfulness was found to be strongly related with neuroticism (negative) followed by conscientiousness (positive). Conscientiousness is the trait of a person who wishes to do one’s work or duty well and thoroughly. Since Langer’s scale measures sociocognitive behavior in terms of novelty seeking, engagement, and novelty producing, we found our hypothesis to be true [63].

Alternatively, mindfulness can be viewed as a mode, or state-like quality, that is maintained only when attention to experience is intentionally cultivated with an open, nonjudgmental orientation to experience [64]. The literature emphasizes the use of meditation as an intervention to improve state mindfulness for psychological well-being (e.g., [18]), which requires an internal intervention of not letting the mind wander and Langer’s nonmeditative (creative) mindfulness perspective of novelty seeking, novelty producing, and engagement in sociocognitive mindfulness, which requires a shift from the psych to the external environment by inducing external stimuli as interventions to respondents, which prompts them to intentionally regulate their mode of thinking and thereby shift or switch from mindlessness to mindfulness.

H2a predicted that undergraduate education would moderate the negative effect of Langer’s mindfulness on the MAAS. Similarly, H2b predicted that undergraduate education would moderate the negative effect of Langer’s mindlessness on the MAAS. We expected that this negative effect would be different for engineers and nonengineers. However, we found that there was no difference in the negative effect of Langer’s mindfulness and mindlessness on the MAAS. In the measurement of MASS and Langer’s manfulness/mindlessness, there was no difference due to education background. Although it was not hypothesized, we also tested the effect of gender, educational background, and MBA specialization on the MAAS and Langer’s mindfulness and mindlessness measurements. Interestingly, we found a difference in the measurement of Langer’s mindlessness due to undergraduate education. However, we did not find an effect of gender, educational background, and MBA specialization on the MAAS and Langer’s measurement. This implies that all the respondents included in this study may have had a similar orientation towards mindfulness/mindlessness due to the similar attitude of MBA students, which was probably due to the homogeneity of the group coming from a similar admission process that involves aptitude tests and interviews. This has a further implication on the mindfulness and mindlessness orientation of the professional groups and the people who are practicing meditation.

In the context of organizations, cultivating the creative mindfulness practices of professionals becomes crucial as the chances of making costly errors are higher, such as with medical nurses, airline staff, heavy vehicle drivers, and traffic control department employees. Further studies are needed for activities where employees act mindlessly when carrying out their routines and trust their gut feeling and skills, such as with construction workers who work at tall heights without safety belts, welders who weld without safety glasses, and phlebotomy technicians who draw blood without hand gloves. In such cases, the professionals assume that paying attention constantly or being conscious of multiple activities/actions may not be possible. Thus, we need to develop fail-proof systems rather than training employees to practice mindful interventions such as meditation to improve state mindfulness and long-term practice to improve the trait of mindfulness. As an implication of our study, we recommend that professionals practice meditative mindfulness to enhance their well-being, as prescribed by [18], or follow instructional interventions such as using technology to draw attention, as suggested by [13] to be mindful. 

### Limitations and Future Research

The main limitation of this study is that the results of this study are only applicable to business school students in India; thus, when the findings of this study are applied to other situations, caution is required. We compared the relationship between the MAAS and Langer scales, but our study but our study did not consider several different measurement scales (FMI, KIMS, SQM, and CAMS). Most of the mindfulness studies were carried out among samples of meditators or for clinical purposes; however, the focus of our study was on nonmeditating business school students. If we had conducted this measurement on meditating students, the results would have been different. 

Future studies can be conducted in different contexts to explore whether the negative effect is applicable or not. These studies can be conducted to compare other different scales; for example, a comparison between the scales that measure state mindfulness and trait mindfulness can be conducted. A similar study can be conducted among a meditating sample group. A longitudinal study can be conducted among various batches with a meditation intervention. 

## Figures and Tables

**Table 1:** Factor loadings and cross loadings.

Items/Factors	MAAS	MFLS	MLLS
MAAS			
I could be experiencing some emotion and not be conscious of it until sometime later.	**0.60**	−0.10	−0.38
I break or spill things because of carelessness, not paying attention, or thinking of something else.	**0.68**	−0.13	−0.40
I find it difficult to stay focused on what’s happening in the present.	**0.72**	−0.25	−0.39
I tend to walk quickly to get where I’m going without paying attention to what I experience along the way.	**0.61**	−0.15	−0.26
I tend not to notice feelings of physical tension or discomfort until they really grab my attention.	**0.68**	−0.19	−0.39
I forget a person’s name almost as soon as I’ve been told it for the first time.	**0.60**	−0.13	−0.26
I am "running on automatic," without much awareness of what I’m doing.	**0.75**	−0.25	−0.42
I rush through activities without being really attentive to them.	**0.81**	−0.30	−0.44
I get so focused on the goal I want to achieve that I lose touch with what I’m doing right now to get there.	**0.59**	−0.04	−0.25
I do jobs or tasks automatically, without being aware of what I’m doing.	**0.82**	−0.23	−0.43
I find myself listening to someone with one ear, doing something else at the same time.	**0.62**	−0.09	−0.36
I drive places on ‘automatic pilot’ and then wonder why I went there.	**0.80**	−0.19	−0.43
I find myself doing things without paying attention.	**0.85**	−0.27	−0.49
I snack without being aware that I’m eating.	**0.73**	−0.09	−0.49
Mindfulness-Langer Scale (MFLS)			
I like to investigate things	−0.08	**0.60**	0.14
I am very creative	−0.18	**0.76**	0.11
I am very curious	−0.20	**0.78**	0.17
I try to think of new ways of doing things	−0.15	**0.84**	0.19
I like to be challenged intellectually	−0.01	**0.58**	0.07
I find it easy to create new and effective ideas	−0.20	**0.74**	0.12
I like to figure out how things work	−0.26	**0.79**	0.17
Mindlessness-Langer Scale (MLLS)			
I avoid thought-provoking conversations (R)	−0.35	0.07	**0.64**
I am rarely aware of changes (R)	−0.39	−0.01	**0.76**
I am rarely alert to new developments (R)	−0.35	0.14	**0.74**
I am not an original thinker (R)	−0.48	0.32	**0.73**

Note: Higher the item score, higher the levels of mindfulness. Values in bold signify that the item’s loading with its respective factors was higher than its loading with other factors. R denotes that these items were reverse coded.

**Table 2:** Reliability and validity testing.

Constructs	Cronbach’s Alpha	Composite Reliability	AVE	Interconstruct Correlations		
				MAAS	MFLS	MLLS
MAAS	0.92	0.93	0.51	**0.71**		
Mindfulness—Langer Scale (MFLS)	0.86	0.89	0.54	−0.25	**0.73**	
Mindlessness—Langer Scale (MLLS)	0.69	0.81	0.52	−0.55	0.20	**0.72**

Note: Values in bold are the square root of AVEs of the respective factor, and they signify that they are higher than the factor’s correlations with other factors.

**Table 3:** Hypotheses testing (structural model).

Hypothesis	Std. Path Coefficients	t-Value	*p*-Value	Hypothesis Supported?
H1a: MFLS --> MAAS	−0.151	2.381	0.017	Yes
H1b: MLLS --> MAAS	−0.524	8.774	0.000	Yes
R^2^	0.329			
Q^2^	0.158			

**Table 4:** Moderation effect of U.G. education background—results of PLS-MGA.

Hypotheses	Independent Variables	Dependent Variable: MAAS						Hypothesis Supported?
		Path Coefficient for U.G. Engineering Group	*p*-Value	Path Coefficient for U.G. Nonengineering Group	*p*-Value	Path Coefficients-Difference	*p*-Value	
H2a	Langer Scale—Mindfulness	−0.182	0.054	−0.177	0.343	−0.005	0.903	No
H2b	Langer Scale—Mindlessness	−0.519	0.000	−0.527	0.000	0.008	0.942	No

**Table 5:** Results of ANOVA (MAAS).

Independent Variables	Dependent Variable: MAAS			
	No. of Respondents	Mean	F-Value	Significance
Gender				
Female	44	2.969		
Male	177	2.994	0.022	0.883
Bachelor’s degree				
Engineering	139	3.018		
Nonengineering	82	2.939	0.336	0.563
MBA Specialization				
Marketing	84	2.943		
Finance	53	3.096		
Operations	40	3.146		
Human resources	20	2.629		
IT and decision sciences	21	2.949		
Strategy	3	2.952	0.922	0.467

**Table 6:** Results of ANOVA (Langer Scale).

Independent Variables	Dependent Variable: Langer Scale—Mindfulness				Dependent Variable: Langer Scale—Mindlessness		
	No. of Respondents	Mean	F-Value	Significance	Mean	F-Value	Significance
Gender							
Female	44	5.233			4.716		
Male	177	5.168	0.204	0.652	4.493	1.218	0.271
Bachelor’s degree							
Engineering	139	5.205			4.416		
Nonengineering	82	5.140	0.297	0.586	4.744	3.914	0.049
MBA Specialization							
Marketing	82	5.321			4.363		
Finance	51	4.958			4.793		
Operations	37	5.172			4.550		
Human resources	20	5.069			4.650		
IT and decision sciences	21	5.232			4.548		
Strategy	3	5.708	1.518	0.185	3.917	1.031	0.400

## Data Availability

Not applicable.
